# The Hidden Risk of Toxoplasmosis in the Expanding Immunomodulated Host Population: A Call for Guidelines and Registries in Patients on Biologics, Small Molecules, and Cellular Therapies

**DOI:** 10.3390/pathogens15070696

**Published:** 2026-06-30

**Authors:** Jose G. Montoya, Stephanie M. Cho, Stephanie Smith, Carlos A. Gomez, Despina G. Contopoulos-Ioannidis

**Affiliations:** 1 Dr Jack S Remington Laboratory for Specialty Diagnostics, United States National Reference Center for Toxoplasmosis, Sutter Health, Palo Alto Medical Foundation, Palo Alto, CA 94301, USA; 2 College of Osteopathic Medicine of the Pacific, Western University of Health Sciences, Pomona, CA 91766, USA; 3 Division of Pediatric Infectious Diseases, Inova Children’s Hospital, Fairfax, VA 22031, USA; 4 Department of Internal Medicine, Division of Infectious Diseases, University of Nebraska Medical Center, Omaha, NE 68198, USA

**Keywords:** toxoplasmosis, *Toxoplasma*, *T. gondii*, targeted immunotherapy, biologics, small molecules, CAR T-cell, perspective, recommendations, guidelines

## Abstract

Targeted immunotherapies with biologics, small molecules, and CAR T-cell therapies have revolutionized treatment across autoimmune, chronic inflammatory, oncologic, and transplant-related conditions. However, they have also expanded the population of patients susceptible to opportunistic infections. *Toxoplasma gondii* (*T. gondii*), a globally prevalent parasite, has emerged as an underrecognized pathogen in this immunomodulated host population. Toxoplasmosis, in such patients, can occur either through reactivation of a chronic/latent/past infection or from an acute/primary infection and may be severe and even fatal. We present here the recommendations for such patients from the Remington Lab, the National Reference Center for Toxoplasmosis in the US. Screening for *Toxoplasma* infections is needed at baseline prior to starting targeted immunotherapy to identify seropositive patients who would benefit from prophylaxis or pre-emptive strategies and seronegative patients who would benefit from measures to prevent primary/acute infections. Prompt diagnosis of *Toxoplasma* disease (toxoplasmosis) with molecular tools (*T. gondii* PCR and/or agnostic metagenomics next-generation sequencing), and prompt initiation of anti-*Toxoplasma* therapy, can be lifesaving and prevent permanent neurocognitive sequelae and vision loss. The immunomodulatory effects of these therapies persist for several months after discontinuation, thereby extending the window of vulnerability. *T. gondii*-seropositive women are at increased risk of vertical transmission, even if targeted immunotherapy was discontinued several months before conception. We make a call for education, guidelines, prospective registries, targeted research, and addition of toxoplasmosis risk in the Warnings section of drug leaflets (and particularly so for *T. gondii*-seropositive women who intend to conceive after having been on targeted immunotherapies).

## 1. Introduction

The expanding use of targeted immunotherapies, including monoclonal antibodies, small molecules, and cellular therapies with chimeric antigen receptor (CAR) T-cells, has created a growing population of immunomodulated hosts who exhibit a transplant-like immune dysfunction. Although targeted immunotherapies biologically induce a narrower immunosuppression state when compared with that observed in conventional transplant and oncologic settings, it has been unequivocally shown that patients on targeted immunotherapies are still at risk of opportunistic infections potentially conveying high morbidity and mortality. In this context, *Toxoplasma gondii* should be recognized as a re-emerging opportunistic pathogen in the non-transplant immunomodulated host. The immunologic perturbations induced by these agents impacting targeted pathways in innate or adaptive immunity, or cytokine signaling, may persist for months after discontinuation, extending the period of vulnerability to severe disease due to reactivation of latent *T. gondii* infection or acute primary infection acquisition.

## 2. Background

In this report from the Remington Laboratory, the National Reference Center for Toxoplasmosis in the US, we would like to bring attention to the risk of toxoplasmosis in patients receiving targeted immunotherapies, particularly in women of reproductive age planning to conceive after having received such therapies, due to the risk for congenital toxoplasmosis. We would like to propose pragmatic strategies for screening, primary prevention, prophylaxis, diagnosis, and management. We would like to also advocate for the following: education (of health care providers and patients about the toxoplasmosis risk associated with these targeted immunotherapies); guidelines (from all subspecialties using targeted immunotherapies for their patients); prospective registries (to better define the incidence and prevalence of toxoplasmosis in the immunomodulated host population); targeted research efforts (to address those questions, including the duration of the persistence of immunomodulation after discontinuation of the targeted immunotherapies); and the addition of toxoplasmosis risk in the “Warnings” section of drug leaflets of these agents (particularly for *T. gondii*-seropositive women who intend to conceive after having been on targeted immunotherapies). The goal is to optimize screening and reduce the preventable morbidity and mortality associated with this opportunistic infection.

In the era of targeted immunotherapy, a new category of vulnerable patient host is defined, the immunomodulated host, whose infection risks arise not from broad or profound cell-mediated immunosuppression, as in traditional solid-organ transplant (SOT) or allogeneic hematopoietic stem-cell transplant (allo-HSCT) recipients, but from a more precise immune modulation, which nevertheless still puts them at risk for opportunistic infections, including toxoplasmosis.

Targeted immunotherapies are now expanded for a wide range of autoimmune diseases, chronic inflammatory conditions, oncologic and transplant related conditions. They are now used by almost all medical subspecialties, including rheumatology, neurology, gastroenterology, dermatology, allergy/immunology, hematology/oncology, transplant medicine, and ophthalmology. Given the ubiquity of *T. gondii* in both high-income and low-income countries, and the expanded use of targeted immunotherapies, the risk of toxoplasmosis in the immunomodulated host population is a worldwide challenge.

Although immune checkpoint inhibitors (ICIs) have not been clearly linked to an increased incidence of toxoplasmosis, consistent with their mechanism of action, as ICIs augment T-cell-mediated effector responses, toxoplasmosis risk may still arise indirectly in such patients when immune-related adverse events (irAEs) develop, necessitating high-dose corticosteroids or other immunosuppressive therapies, thereby creating a transient state of secondary immunosuppression [1].

A 2025 systematic review on this topic from our team at the Remington Lab [2] and a 2022 review and data from the French National Reference Center for Toxoplasmosis [3] identified 55 toxoplasmosis cases in patients receiving targeted immunotherapies, including two congenital toxoplasmosis cases, associated with 20 distinct immunomodulators, encompassing nearly all major classes of targeted immunotherapies with biologics, small molecules and cellular CAR T-cell therapies (Table 1). The reported toxoplasmosis cases originated from 18 different countries across multiple continents, including the US and several European countries, underscoring the global nature of this emerging clinical concern.

## 3. Clinical Manifestations

**Figure 1:**
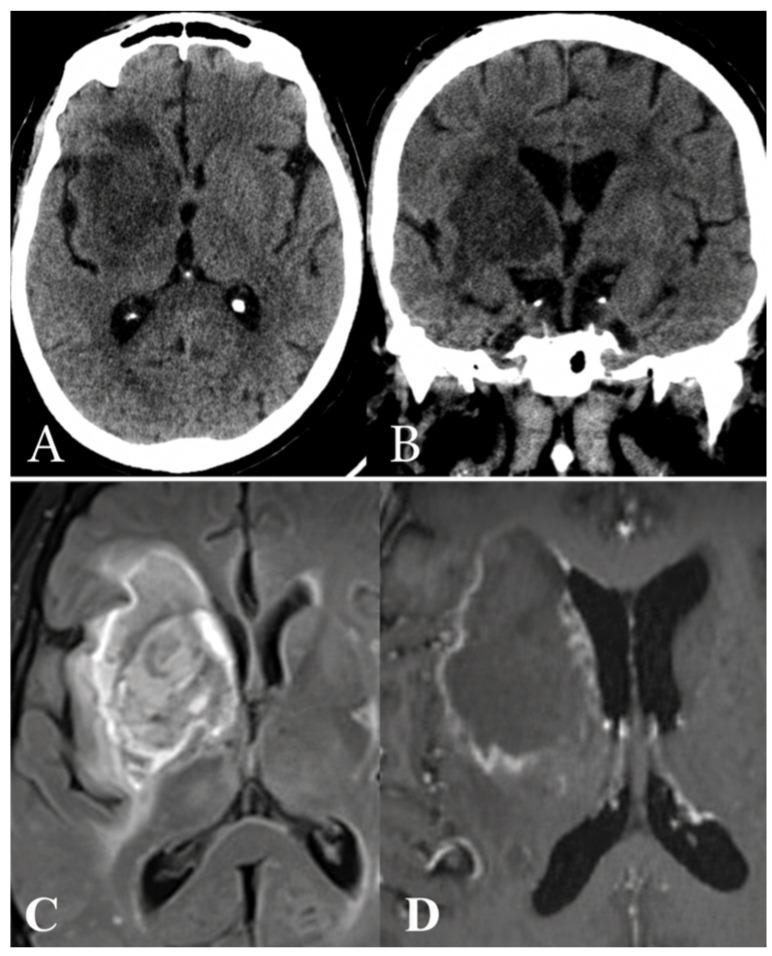
Case of reactivation of latent *Toxoplasma* infection on adalimumab [4]: In a 62-year-old female with treatment refractory rheumatoid arthritis, adalimumab was added to her treatment regimen two months prior to presentation. She presented with a two-week history of worsening temporal disorientation, unsteady gait, left hemiparesis and associated lymphopenia. Brain CT showed a large hypodense space occupying mass lesion in the right basal ganglia, surrounded by edema involving the frontal and temporal lobes and mass effect with effacement of cerebral sulci, compression of the ventricular system and mild left deviation of midline structures (**A**,**B**). MRI showed the mass lesion was heterogeneous on T2/FLAIR with ring-enhancement with gadolinium (**C**,**D**). *T. gondii* serology suggested reactivation of latent infection. Brain biopsy showed necrosis and the presence of bradyzoites and free tachyzoites with positive *T. gondii* immunohistochemistry, establishing the diagnosis of cerebral toxoplasmosis. Adalimumab was discontinued and the patient was successfully treated with pyrimethamine/sulfadiazine/folinic acid. At 6 months follow up, patient had complete recovery. (Reproduced with permission from de Almeida GB et al. Cerebral Toxoplasmosis as an Uncommon Complication of Biologic Therapy for Rheumatoid Arthritis: Case Report and Review of the Literature. *Brain Sciences*, Aug 8 2022;12(8), doi:10.3390/brainsci12081050) [4].

**Figure 2:**
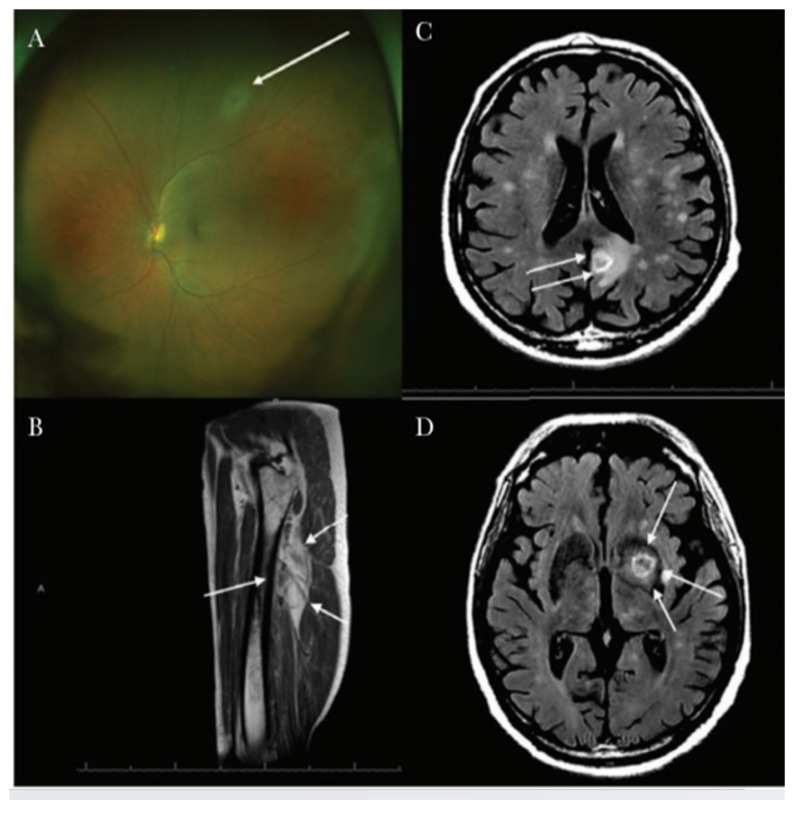
Case of disseminated toxoplasmosis on trametinib after acute *Toxoplasma* infection [5]: A 65-year-old male with rheumatoid arthritis and right orbit pseudolymphoma, required the addition of trametinib due to disease progression despite multimodal therapy. Five weeks later, he developed gait incoordination and fine motor skills deficits. The patient had CSF lymphocytic pleocytosis, retinitis ((**A**) shows left eye fundus with retinal whitening in the superior area consistent with retinitis [white arrow] without overlying vitritis on exam), myocarditis and myositis ((**B**) shows T1 MRI image of the right thigh consistent with myositis, with increased fluid signal in vastus intermedialis muscle [white arrows]). Brain MRI (T2 FLAIR [fluid-attenuated inversion recovery] images) showed post contrast, multiple ring enhancing lesions of toxoplasmosis [white arrows] (**C**,**D**). Serology suggested acute *Toxoplasma* infection. The patient reported recent consumption of wild boar sausages while on trametinib. After a 16-week course of high dose TMP-SMX, the patient had substantial clinical improvement; at 4 months follow up, there was a reduction in size of the CNS lesions. (Reproduced with permission from Gharamti AA et al. Acute *Toxoplasma* Dissemination with Encephalitis in the Era of Biological Therapies. *Open Forum Infectious Diseases*, Nov 2018; 5(11):ofy259, doi:10.1093/ofid/ofy259) [5].

In the immunomodulated host, toxoplasmosis can be severe and life-threatening, and can present with cerebral toxoplasmosis [3,4,5,6,7,8], disseminated disease [5,6,7,8,9], pneumonic toxoplasmosis complicated by respiratory failure [9], and/or ocular toxoplasmosis with permanent vision loss [10,11,12], among other manifestations (Table 1, Figure 1 and Figure 2). Toxoplasmosis can develop from reactivation of a latent/chronic/past infection or from a primary/acute infection; both types of infections can lead to severe disease. Over one-third of toxoplasmosis cases in patients on targeted immunotherapies can have unfavorable outcomes, including fatal outcomes due to toxoplasmosis [9,13,14,15], permanent neurocognitive sequelae [16], or permanent vision loss [10,11,12].

**Table 1:** Reported toxoplasmosis clinical manifestations by targeted immunotherapies with biologics, small molecules, and CAR-T cell therapies (e.g., anti-CD20, anti-CD40, anti-CD52, anti-IL12/IL23, anti-IL7A, integrase inhibitors, TNF inhibitors, T-cell co-stimulation inhibitors, CAR T-cells, JAK inhibitors, MEK inhibitors, tyrosinase kinase inhibitors, and sphingosine 1-phosphate receptor modulators) [2].

Targeted Immunotherapy	Drug Class	Toxoplasmosis Clinical Manifestations
***Biologics***		
Abatacept	T-cell co-stimulation inhibitor	Ocular toxoplasmosis [17]
Adalimumab	TNF-a inhibitor	Mother-to-child transmission/severe fetal congenital toxoplasmosis [18], Cerebral toxoplasmosis [4,6,19], Ocular toxoplasmosis [12,20,21,22,23,24], Lymphadenopathy, Malaise [25]
Alemtuzumab	Anti-CD52 inhibitor	Cerebral toxoplasmosis [15,26], Death [15]
Anakinra	Interleukin-1 receptor inhibitor	Ocular toxoplasmosis [3]
Belatacept	T-cell co-stimulation inhibitor	Cerebral toxoplasmosis [27], Ocular toxoplasmosis [28]
Etanercept	TNF-a and b inhibitor	Ocular toxoplasmosis [3,10]
Golimumab	TNF-a inhibitor	Cerebral toxoplasmosis [14], Death [9]
Infliximab	TNF-a inhibitor	Mother-to-child transmission/congenital toxoplasmosis [29], Cerebral toxoplasmosis [30,31,32], Ocular toxoplasmosis [10], Mildly symptomatic [3]
Iscalimab	Anti-CD40 inhibitor	Disseminated toxoplasmosis [33]
Ixekizumab	Anti-IL-7A inhibitor	Lymphadenopathy [34]
Natalizumab	Integrase inhibitor	Ocular toxoplasmosis [35]
Rituximab	Anti-CD20 inhibitor	Death [13] Cerebral toxoplasmosis [16,36,37,38,39], Ocular toxoplasmosis [3]
TNF-a inhibitor (not reported)	TNF-a inhibitor	Cerebral toxoplasmosis [3,39]
Ustekinumab	Anti-IL12/IL23 inhibitor	Ocular toxoplasmosis [11], Lymphadenopathy [40]
***Cellular Therapies***		
CAR T-cells	CAR T-cells	Cerebral toxoplasmosis [41,42]
***Small molecules***		
Erlotinib	Tyrosine kinase inhibitor	Ocular toxoplasmosis [43]
Fingolimod	Sphingosine 1-phosphate receptor modulator	Cerebral toxoplasmosis [44]
Imatinib	Tyrosine kinase inhibitor	Ocular toxoplasmosis [45]
JAK 3 inhibitor (not reported)	JAK 3 inhibitor	Pneumonic toxoplasmosis [46]
Ruxolitinib	JAK inhibitor	Cerebral toxoplasmosis [47,48], Ocular toxoplasmosis [49]
Trametinib	MEK inhibitor	Disseminated toxoplasmosis (cerebral, ocular, myocarditis, myositis) [5]

*Abbreviations:* CAR T-cell: chimeric antigen receptor T-cell, IL-interleukin, JAK: janus kinase, MEK: mitogen-activated extracellular signal regulated kinase, TNF: tumor necrosis factor. *Footnote:* 1. Without prospective registries, the denominator for these toxoplasmosis cases remains unknown. 2. However, in the absence of large prospective registries and targeted research efforts to address the incidence and prevalence of toxoplasmosis in the immunomodulated host population and the duration of immunomodulation after having been on targeted immunotherapies, the findings from our systematic review [2] (Cho et al *Pathogens* 2025) and from the French Registry [3] (Durieux et al *Plos Negl Trop Dis* 2022) on this topic (55 cases of toxoplasmosis associated with 20 biologics/targeted immunotherapies across 18 countries, including two cases of congenital toxoplasmosis) lead us to believe that these cases likely represent only the tip of the iceberg. It is important both for the health care providers and the patients to know about those cases (e.g., through warnings in the drug leaflets) so that toxoplasmosis will be promptly considered in the differential diagnosis, should compatible clinical signs and symptoms develop in patients on targeted immunotherapies. 3. Although ocular toxoplasmosis reactivation can also occur in the immunocompetent host, in the setting of targeted immunotherapy, it is a reflection of immunomodulation.

Another major concern is the vertical transmission of congenital toxoplasmosis in *T. gondii*-seropositive pregnant women who had been on targeted immunotherapies, even several months before conception. Two cases of congenital toxoplasmosis have been described in the literature, both originating from Switzerland [18,29]. The first case occurred in an infant born to a mother with a history of chronic or past *T. gondii* infection (likely acquired 6–7 months before conception), who was receiving adalimumab for ankylosing spondylitis until up to 5 months before conception. In this case, mother-to-child-transmission and fatal congenital toxoplasmosis occurred, with cerebral and disseminated disease, despite discontinuation of adalimumab 5 months before conception [18]. The second case occurred in an infant born to a *T. gondii*-seropositive mother, who had continued infliximab for Crohn’s disease during gestation, given every 8 weeks except for the third trimester, along with azathioprine [29]. The mother had serologic evidence of chronic/past *T. gondii* infection early in gestation with high *T. gondii* IgG avidity. Vertical transmission and symptomatic congenital toxoplasmosis developed, presumably from reactivation of the chronic/latent past infection, with neonatal fever, persistent tachycardia, tachypnea, hypoxia, poor peripheral perfusion, B cell lymphopenia, and hypogammaglobulinemia.

## 4. Magnitude of the Problem: Increasing Immunomodulated Host Population

Targeted immunotherapies are now recommended for a wide range of conditions. In the United States, more than 28 million individuals have asthma, over 1.5 million have rheumatologic diseases, and more than 1.8 million new cancer cases are diagnosed annually. Between 2003 and 2013, among 2.01 million asthmatic patients in the US, 6651 (0.3%) received biologic therapy such as benralizumab, dupilumab, mepolizumab, omalizumab, or reslizumab [50].

Moreover, a Medicare claims study of prescriptions for biologics for rheumatologic conditions (tofacitinib, baricitinib, upadacitinib, tocilizumab, sarilumab, abatacept) in the US over a 10-year period (2013–2022) identified 1.5 million such prescriptions [51]. Furthermore, the oncology segment holds a 29.4% market share in the biologic market in the US [52].

Considering that, in the US, approximately 11% of the population greater than 6 years old is seropositive for *T. gondii*, one can extrapolate that, each year in the US, several hundreds of *T. gondii*-seropositive individuals on biologics are at risk for toxoplasmosis [53].

The number of biologics and small molecules approved each year is increasing exponentially. For instance, in 2024, 32 new small molecules were approved; with oncology receiving the highest number of approvals, followed by dermatology, hematology, and cardiovascular conditions [54]. The expansion of targeted therapies amplifies the population at risk for opportunistic infections, such as toxoplasmosis, highlighting a need for mitigating strategies across diverse clinical disciplines.

## 5. Prevention

In the absence of robust evidence for the true incidence and prevalence of toxoplasmosis in the immunomodulate host population, we would like to *err* on the side of caution and make recommendations and call for improved education, guidelines from all subspecialties, prospective registries, targeted research efforts, and the addition of toxoplasmosis risk in the “Warnings” section of product inserts (drug leaflets). Health care providers and patients need to be educated to promptly consider toxoplasmosis should compatible clinical signs and symptoms develop. In particular, *T. gondii*-seropositive women of reproductive age planning to become pregnant after having been on these agents should be clearly informed about the risk of congenital toxoplasmosis.

The Remington Lab recommends the following measures: (a) All patients scheduled to receive targeted immunotherapies should undergo baseline serologic screening for *Toxoplasma* IgG and IgM prior to starting the immunotherapy (along with screening for other relevant opportunistic pathogens); (b) *Toxoplasma* IgG-seropositive patients should undergo a baseline ophthalmologic evaluation, for identification of possible old chorioretinal scars that could reactivate during targeted immunotherapy and cause vision threatening lesions; (c) *Toxoplasma* IgG-seropositive patients should be considered for primary prophylaxis and/or a preemptive PCR strategy (and particularly those receiving combination immunotherapies or prolonged regimens); (d) Close clinical monitoring for signs and symptoms of toxoplasmosis reactivation and prompt molecular laboratory testing (e.g., blood *Toxoplasma* PCR or next generation sequencing metagenomics tests) should be used if compatible clinical syndromes develop; and (e) Serologic screening for toxoplasmosis (*Toxoplasma* disease) while a patient is already receiving a targeted immunotherapy may be misleading. Although a positive *Toxoplasma* IgG remains informative, a negative IgG may be falsely negative.

Recommendations for *T. gondii*-seropositive women of reproductive age planning to become pregnant after having been on targeted immunotherapies are listed separately below.

Education to prevent acute *Toxoplasma* infections in seronegative patients through avoidance of exposure risks is critical [55]. Failure to provide timely education on toxoplasmosis risk factors can result in serious outcomes. In the 2025 systematic review by the Remington lab [2], cases of severe toxoplasmosis were identified from acute infections after food exposure, linked to the consumption of wild boar sausages [5] or undercooked meat [28], including wild game meat, while on targeted immunotherapies [2,43]. Such cases included patients with disseminated disease or vision-threatening ocular infections. Clinicians should routinely counsel such patients on avoiding exposure to *T. gondii* from infected/contaminated food, untreated water, and soil contact, for the duration of targeted immunotherapy and at least 6 months after its discontinuation [55].

Furthermore, patients on targeted immunotherapies should be educated about toxoplasmosis signs and symptoms and the need to seek immediate medical attention should unexplained fever, fatigue, neuropsychiatric symptoms, focal neurologic signs and symptoms, ocular symptoms, lymphadenopathy, pneumonia, myocarditis, myositis, or hepatitis develop, as these could represent severe toxoplasmosis [1].

## 6. Special Pregnancy Considerations for Women of Reproductive Age on Targeted Immunotherapies

Women who plan to conceive after having been on targeted immunotherapies for autoimmune diseases or chronic inflammatory conditions should consult with an infectious diseases specialist with expertise in toxoplasmosis in an immunomodulated host and congenital infections. For decades, immunocompetent women with chronic/latent past *Toxoplasma* infection acquired months to years before conception have been regarded as not being at risk for congenital toxoplasmosis. However, this concept may not apply for *T. gondii*-seropositive women who have been on targeted immunotherapies, even several months before conception, as the immune dysregulation/immunomodulation associated with targeted immunotherapy can persist for more than 6 months after treatment discontinuation, increasing the risk of vertical transmission of congenital toxoplasmosis [18].

Moreover, changes in maternal immunologic environment during a normal pregnancy may lead to reactivation of prior latent ocular toxoplasmosis; a phenomenon reported in 9% to 20% of *T. gondii*-seropositive pregnant women, which can further increase the risk of vertical transmission to the fetus [56,57]. Cases of congenital toxoplasmosis have been reported in *T. gondii*-seropositive pregnant women after reactivation of their ocular toxoplasmosis during pregnancy [58]. This risk of vertical transmission in the setting of ocular disease reactivation during pregnancy may be further increased in *T. gondii*-seropositive pregnant women who have been on targeted immunotherapies. Thus, preconception eye examination in such women is of particular importance worldwide.

The Remington Lab recommends the following measures for women who have been on targeted immunotherapies and plan to conceive: (a) mandatory routine prenatal screening for *Toxoplasma* infection of all such women with *Toxoplasma* IgG and IgM; (b) education to avoid exposure risks (via food and/or activities) for acute primary *Toxoplasma* infection [24] during gestation and follow serological testing throughout gestation in an attempt to promptly diagnose possible acute infection (via detection of seroconversion); (c) for *T. gondii*-seropositive women, delay of pregnancy for at least 12 months after discontinuation of the targeted immunotherapy (the duration of the immunomodulation may be prolonged, even after discontinuation of the immunotherapy); (d) for *T. gondii*-seropositive women, screening with blood *Toxoplasma* PCR prior to becoming pregnant to exclude active parasitemia; (e) for *T. gondii*-seropositive women, ophthalmologic evaluation to detect old retinal scars (that could reactivate during pregnancy, possibly increasing the risk of vertical transmission); and (f) for *T. gondii*-seropositive women, spiramycin suppressive therapy throughout their future pregnancy to prevent transplacental vertical transmission (if pregnancy occurs within 12 months from targeted immunotherapy discontinuation, or while on targeted immunotherapy [if targeted immunotherapy is continued during pregnancy, despite the increased risk of congenital toxoplasmosis]).

Particular attention is needed, in the proper counseling of women who intend to conceive, regarding the risks associated with the continuation of targeted immunotherapy during gestation for those who are *T. gondii*-seropositive. *T. gondii*-seropositive pregnant women who have remained on targeted immunotherapies during gestation are at increased risk for vertical transmission of congenital toxoplasmosis [29]. The Remington Lab recommends that women with chronic autoimmune diseases of reproductive age on targeted immunotherapies, who are found to be *T. gondii*-seropositive before pregnancy, should not be offered (if feasible) the option of continuing the immunomodulators during gestation due to the increased risk of congenital toxoplasmosis. As above, pregnancy should be delayed for at least 12 months after discontinuation of the targeted immunotherapy.

## 7. Diagnosis of Toxoplasmosis (*Toxoplasma* Disease)

The timely diagnosis and treatment of toxoplasmosis (*Toxoplasma* disease) in patients on targeted immunotherapies can be lifesaving. Clinicians should maintain a high index of suspicion for toxoplasmosis in both *Toxoplasma* seropositive and seronegative patients who are on targeted immunotherapies and who present with compatible clinical syndromes. Severe toxoplasmosis in such patients can result both from reactivation of chronic/latent (past) infections and from acute primary infections.

Reactivation of a chronic/latent past *Toxoplasma* infection should be suspected if a compatible clinical syndrome occurs within a few to several months from initiation or dose escalation of targeted immunotherapy, reflecting the delayed onset of immune suppression associated with these agents. Active toxoplasmosis several years after the initiation of a targeted immunotherapy should raise the suspicion of an acute primary *Toxoplasma* infection. Breakthrough infections may also occur, even on adequate anti-*Toxoplasma* prophylaxis [15].

Serologic screening for toxoplasmosis in patients who are already on targeted immunomodulators, particularly those with lymphopenia, may be misleading. A positive *Toxoplasma* IgG is informative; however, a negative *Toxoplasma* IgG may be falsely negative due to the lymphopenia from the immunomodulation. In those cases, only molecular diagnostics can be informative.

## 8. Molecular Diagnostics (Targeted and Agnostics Metagenomics)

In the appropriate clinical settings, diagnostic evaluation of toxoplasmosis (*Toxoplasma* disease) should prioritize molecular-based testing, including *Toxoplasma* PCR from relevant specimens such as whole blood or buffy coat, bronchoalveolar lavage (BAL), cerebrospinal fluid (CSF), urine, vitreous or aqueous fluid, and other involved body sites. Agnostic metagenomic next-generation sequencing (mNGS) can also be particularly valuable in the immunomodulated hosts, where the differential diagnosis is often broad and conventional microbiologic testing may be inconclusive. In the United States, commercially available plasma-based mNGS assays include the Karius test (Karius Inc, Redwood City, CA, USA), while in Europe, similar testing is available through the DISQVER platform (Noscendo GmbH, Germany) [59]. For CSF, agnostic mNGS testing is also commercially available (e.g., DelveBio, Boston, MA, USA) [60]. mNGS enables early pathogen identification and initiation of lifesaving therapy, particularly when clinical presentation is atypical or when standard microbiologic diagnostics are uninformative. Indeed, interpretation of *Toxoplasma* serologies can be limited by lymphopenia, transfusions, or IVIG. Moreover, *Toxoplasma*-positive IgM test results should always be confirmed at a reference laboratory (e.g., in the United States, the JS Remington Laboratory for Specialty Diagnostics, Palo Alto, CA) [61].

## 9. Management of Toxoplasmosis (*Toxoplasma* Disease)

Prompt initiation of anti-*Toxoplasma* treatment is critical to prevent severe irreversible sequelae or death. First-line therapy consists of either combination treatment of pyrimethamine, sulfadiazine, and folinic acid, or a course of trimethoprim-sulfamethoxazole [2]. The duration of anti-*Toxoplasma* therapy should be 6–8 weeks; however, longer therapy (up to 3–4 months) may be needed in some cases with delayed response (e.g., severe ocular disease). Modification of the targeted immunotherapy may also be needed and should be conducted in consultation with the primary specialty team and infectious diseases experts. Discontinuation of targeted immunotherapy may be required in some severe cases such as disseminated disease, cerebral toxoplasmosis, pneumonic toxoplasmosis with respiratory failure and/or ocular toxoplasmosis in a vision-threatening area [2]. Secondary prophylaxis/suppressive therapy after resolution of the active toxoplasmosis should be considered, especially in patients who remain deeply immunomodulated or who require re-initiation of targeted immunotherapy [2].

## 10. Registries

Although several registries for patients on biologics already exist [62,63,64,65,66,67], we recommend the establishment of dedicated registries to capture toxoplasmosis (among other opportunistic infections) as platforms for ongoing post-marketing safety surveillance. The toxoplasmosis risk should be incorporated into drug-safety labeling and prescribing information, including patient and provider information leaflets. A special warning should also be added for women of reproductive age for the risk of congenital toxoplasmosis in *T. gondii*-seropositive women on targeted immunotherapies during pregnancy and up to 12 months before conception.

## 11. Conclusions

As the use of targeted immunotherapy expands, the population of the immunomodulated host at risk for toxoplasmosis (and other opportunistic infections thereof) will inevitably grow. The 55 toxoplasmosis cases identified in the 2025 systematic review from the Remington Lab, the National Reference Center for Toxoplasmosis in the US, and the 2022 review and report from the French National Reference Center for toxoplasmosis, in patients on targeted immunotherapies with biologics, small molecules, or cellular therapies represents only the visible fraction of a much larger, unrecognized global burden. Toxoplasmosis may remain undiagnosed without clinical suspicion, appropriate molecular testing, including agnostic testing with metagenomics next generation sequencing approaches, and/or postmortem examination.

Education of health care providers and patients, proactive risk assessment, enhanced agnostic diagnostic approaches, and toxoplasmosis registries (among other opportunistic infections) for patients on targeted immunotherapies are critical steps to identify, prevent, and manage toxoplasmosis in the emerging immunomodulated host population.

Decades of experience at the Remington Lab witnessing the severe consequences of toxoplasmosis in patients across the United States compelled us to summarize the toxoplasmosis risk for this exponentially increasing immunomodulated host population. It is a risk that current medicine may be underrecognizing—and for which patients may be paying the price. Ongoing research is needed to further refine recommendations as more experience accumulates. Registries are an excellent foundation, but they should be complemented by targeted research efforts.

## Data Availability

All data for this project are available in this paper.
